# Routine Hematological Parameters May Be Predictors of COVID-19 Severity

**DOI:** 10.3389/fmed.2021.682843

**Published:** 2021-07-16

**Authors:** Paulina B. Szklanna, Haidar Altaie, Shane P. Comer, Sarah Cullivan, Sarah Kelliher, Luisa Weiss, John Curran, Emmet Dowling, Katherine M. A. O'Reilly, Aoife G. Cotter, Brian Marsh, Sean Gaine, Nick Power, Áine Lennon, Brian McCullagh, Fionnuala Ní Áinle, Barry Kevane, Patricia B. Maguire

**Affiliations:** ^1^Conway SPHERE Research Group, Conway Institute, University College Dublin, Dublin, Ireland; ^2^School of Biomolecular and Biomedical Science, University College Dublin, Dublin, Ireland; ^3^SAS UK Headquarters, Wittington House, Buckinghamshire, United Kingdom; ^4^Department of Respiratory Medicine, Mater Misericordiae University Hospital, Dublin, Ireland; ^5^Department of Haematology, Mater Misericordiae University Hospital, Dublin, Ireland; ^6^SAS Institute Ltd., La Touche House, Dublin, Ireland; ^7^School of Medicine, University College Dublin, Dublin, Ireland; ^8^UCD Centre for Experimental Pathogen and Host Research, Dublin, Ireland; ^9^Department of Infectious Diseases, Mater Misericordiae University Hospital, Dublin, Ireland; ^10^Department of Critical Care Medicine, Mater Misericordiae University Hospital, Dublin, Ireland; ^11^Department of Haematology, Rotunda Hospital, Dublin, Ireland; ^12^UCD Institute for Discovery, University College Dublin, Dublin, Ireland

**Keywords:** COVID-19, critical care, machine learning, AI in healthcare, blood, hematological parameters, activated partial thromboplastin time, platelet count

## Abstract

To date, coronavirus disease 2019 (COVID-19) has affected over 100 million people globally. COVID-19 can present with a variety of different symptoms leading to manifestation of disease ranging from mild cases to a life-threatening condition requiring critical care-level support. At present, a rapid prediction of disease severity and critical care requirement in COVID-19 patients, in early stages of disease, remains an unmet challenge. Therefore, we assessed whether parameters from a routine clinical hematology workup, at the time of hospital admission, can be valuable predictors of COVID-19 severity and the requirement for critical care. Hematological data from the day of hospital admission (day of positive COVID-19 test) for patients with severe COVID-19 disease (requiring critical care during illness) and patients with non-severe disease (not requiring critical care) were acquired. The data were amalgamated and cleaned and modeling was performed. Using a decision tree model, we demonstrated that routine clinical hematology parameters are important predictors of COVID-19 severity. This proof-of-concept study shows that a combination of activated partial thromboplastin time, white cell count-to-neutrophil ratio, and platelet count can predict subsequent severity of COVID-19 with high sensitivity and specificity (area under ROC 0.9956) at the time of the patient's hospital admission. These data, pending further validation, indicate that a decision tree model with hematological parameters could potentially form the basis for a rapid risk stratification tool that predicts COVID-19 severity in hospitalized patients.

## Introduction

The novel severe acute respiratory syndrome coronavirus 2 (SARS-CoV-2), causing the Coronavirus disease 2019 (COVID-19), has affected over 100 million globally with over 2.5 million fatalities to date leading to a global pandemic of unprecedented size (1). COVID-19 symptoms can vary from person to person, leading to a clinical manifestation of disease ranging from asymptomatic to mild infections, through to serious, life-threatening cases requiring admission to the intensive care unit (ICU) (2, 3). The severity of COVID-19 depends on several factors including age, gender, and the presence of existing comorbidities such as diabetes, hypertension, or respiratory disease (4–6); however, it is difficult to predict the future severity of COVID-19 infection at the time of the patients' admission to the hospital.

Hospitalized COVID-19 patients receive various care regimens. These regimens depend on the severity of COVID-19 and result in a varying rate of ICU admissions (7–9). Decisions regarding the best care regimen for individual patients are challenging and require specialized clinical expertise and effective, inter-departmental communication (10–13). Several COVID-19 specific risk scores have been designed to support clinical decision making and facilitation of appropriate care. These utilize various combinations of patient characteristics, physiological parameters (14), biochemical parameters (15), and radiological features (16, 17). However, these processes can be time consuming and require extensive clinical patient evaluation utilizing advanced techniques by highly experienced personnel. The ability to quickly diagnose and identify patients who would benefit from early, invasive treatment measures at the time of hospital admission, for example, is of critical importance and would be of substantial value (18).

A vast amount of information/data is generated for each hospitalized patient and machine learning (ML) can be used to extract important insights (19). For example, recent utilization of ML in the healthcare setting has enabled more accurate diagnoses, by querying electronic health records to determine the deterioration risk in patients with acute kidney injury (20), or the likelihood of acute myocardial infarction (21). Interestingly, artificial intelligence (AI) has also been used to indicate the risk of future diseases in children and survival in burn patients (22–24). ML and AI technologies have also been utilized to aid decision making during the COVID-19 pandemic including combining data from computer tomography (CT) scans together with clinical symptoms, laboratory testing, and exposure history to create an AI system for the rapid diagnosis of COVID-19 (25). Moreover, a decision tree model has been used to identify a set of certain features (lactate dehydrogenase, lymphocyte proportion, and high-sensitivity C-reactive protein) to predict mortality in hospitalized COVID-19 patients with 97% accuracy (26). Although several ML models have recently been proposed for the prediction of ICU admission among COVID-19 patients and may be useful in resource management in the hospital setting, these models require extensive patient information such as CT scans (27, 28), other imaging data (29, 30), extensive specialist knowledge, that is, APACHE II score (31), additional protein marker tests (32), or extensive patient history and clinical workup (33, 34). Various implementation barriers (such as the creation of robust infrastructure, appropriate staff training, and quality control of the biophysical biomarkers) would have to be overcome to successfully implement these models in a busy clinical setting. As a result, an easily accessible and interpretable model for the prediction of severity of COVID-19, based on routinely obtained clinical parameters, remains an urgent, unmet clinical challenge. Due to this necessity, the aim of the present study was to determine if hematological parameters, which are routinely obtained for every patient in a hospital setting, can be used as predictors for severity and progression of COVID-19 infection.

## Methods

Data are presented from a single-center retrospective cohort study during the first wave of COVID-19 in Ireland (March to May 2020) at the Mater Misericordiae University Hospital (MMUH), Dublin, Ireland. Ethical approval was granted by the Institutional Review Board of the MMUH (1/378/2077). Anonymized datasets describing limited baseline clinical factors (age, gender, and outcome) and hematological laboratory parameters among hospitalized patients with severe COVID-19 (requiring critical care support; *n* = 34) and non-severe COVID-19 (not requiring critical care; *n* = 20) were compiled from routine clinical testing results. Access to detailed clinical factors such as baseline characteristics and treatment were unavailable due to the data protection regulation. SARS-CoV-2 infection was confirmed in all cases by RT-PCR analysis of nasopharyngeal swab specimens. Of the severe COVID-19 patients (*n* = 34), 24% (*n* = 8) were an inpatient at the time of dataset access, 20% (*n* = 7) had passed away, and 56% (*n* = 19) had been discharged from the hospital. Of the non-severe COVID-19 patients, 15% (*n* = 3) were an inpatient at the time of dataset access, 0% (*n* = 0) had passed away, and 75% (*n* = 15) had been discharged from the hospital. For the remaining 10% (*n* = 2), no outcome was recorded.

### Predictors and Outcome

Routine clinical hematology laboratory data obtained on the day of patients' positive COVID-19 test were utilized as candidate predictors for the model. The full list of parameters assessed can be found in Supplementary Table 1. The outcome recorded for each patient was the need for subsequent ICU care during hospitalization.

### Data Pre-processing and Model Development

The hematological data for prediction of severe COVID-19 infection were obtained from patients on the day of positive RT-PCR result for SARS-CoV-2 (at the time of hospital admission). Hematological data from 54 patients from two cohorts, severe and non-severe COVID-19, were uploaded into SAS Viya (version 3.5) and pre-processed before model building. In brief, data were amalgamated and cleaned, variables with >50% missing data were rejected, and mean imputation was used for other variables with missing data. Specifically, imputation was performed for the variables mean platelet volume (MPV), platelet count-to-MPV ratio (PLT_MPV), activated partial thromboplastin time (aPTT), fibrinogen, and D-dimer.

A decision tree model was developed in SAS Viya based on the 54 observations. Latin Hypercube Sampling was used to hyper parameterize the decision tree, and the following criteria were selected: maximum branches: 2, maximum depth: 5, and leaf size: 6. The performance of the model was assessed using an area under receiver operating characteristic (AUROC). In addition, sensitivity and specificity for the model were calculated.

## Results

**Figure 1:**
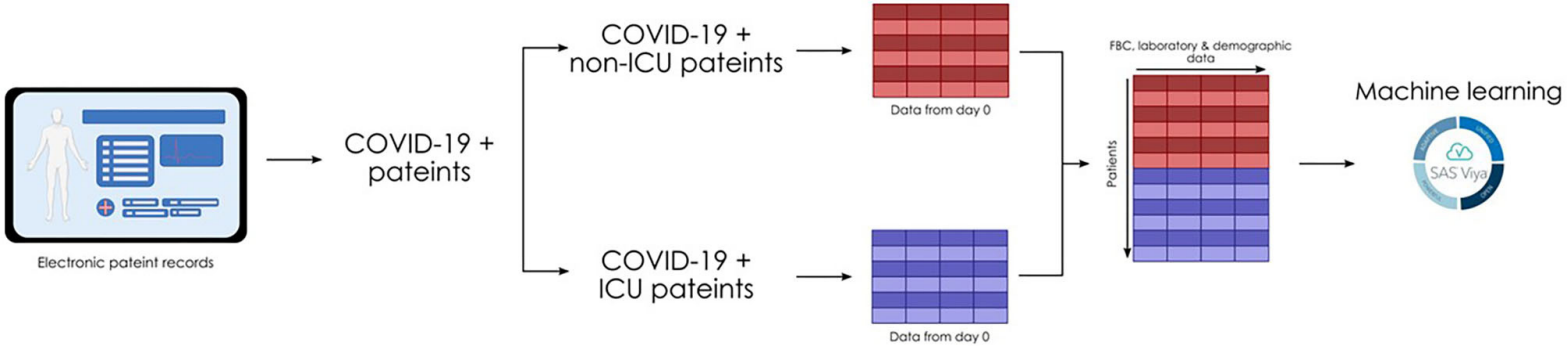
A schematic of patient data collection for decision modeling. Data of COVID-19-positive patients were extracted retrospectively from a central database. Data from the day of positive swab was extracted from severe COVID-19 patients (requiring critical care during their hospital stay) and non-severe COVID-19 patients (not requiring critical care during their hospital stay). Data were amalgamated, pre-processed, and subjected to machine learning.

Anonymized data pertaining to 36 patients with COVID-19 requiring critical care level support from March to May 2020 at MMUH, Dublin, Ireland, were collected. Of the 36 severe COVID-19 patients, 34 were included in the analysis (two patients were excluded as the positive SARS-CoV-2 RT-PCR test was obtained after ICU admission), together with a control group of 20 non-severe patients with COVID-19 (Figure 1). Patients' clinical characteristics at the day of positive COVID-19 swab are presented in Table 1. Within our study groups, non-severe COVID-19 patients were significantly older (69.25 ± 17.1 years old; *p* = 0.031) than the severe COVID-19 patients (59.4 ± 10.5 years old). This finding, contradicting existing literature (35, 36), can be a result of the approach to fight COVID-19, taken at the start of the pandemic in Ireland, where younger patients with fewer comorbidities were prioritized for critical care. The ratio of males to females was similar for both groups with 50 and 62% males in non-severe and severe COVID-19 groups, respectively. Furthermore, we have observed that the activated partial thromboplastin time (aPT) (29.22 ± 1.99 s in the non-severe group; 33.08 ± 8.35 s in the severe group; *p* = 0.047) and D-dimer levels (1.01 ± 0.75 mg/L in the non-severe group; 4.95 ± 6.36 mg/L in the severe group; *p* = 0.0043) were significantly increased in our severe COVID-19 cohort (Table 1). Both of these findings were previously reported by others (8, 37, 38). We found no difference in patients' prothrombin time (PT) and fibrinogen levels between groups (Table 1).

**Table 1:** Clinical characteristics of non-severe and severe patients with confirmed COVID-19 on admission.

	**Non-severe**	**Severe**	***P*-value**
	**COVID-19**	**COVID-19**	
	**(*n* = 20)**	**(*n* = 34)**	
Age (years)	69.25 ± 17.7	59.4 ± 10.5	0.031^*^
Patients >60 years old, *n* (%)	10 (50%)	16 (47%)	1^§^
Male, *n* (%)	10 (50%)	21 (62%)	0.57^§^
PCT (%)	0.246 ± 0.10	N/A	N/A
P-LCR (%)	23.43 ± 7.57	N/A	N/A
PT (s) (10.4–13.0)	13.26 ± 2.02	13.87± 5.47	0.5603^*^
aPTT (s) (25.0–36.5)	29.22 ± 1.99	33.08± 8.35	0.047^*^
Fibrinogen (g/L) (1.5–4.0)	4.65 ± 1.64	5.58 ± 1.74	0.1973^*^
D-Dimer (mg/L) (0.0–0.5)	1.01 ± 0.75	4.95 ± 6.36	0.0043^*^

*PCT, plateletcrit; PLCR, platelet–large cell ratio; PT, prothrombin time; aPTT, activated partial thromboplastin time*.

*Reference ranges of PT, aPTT, fibrinogen, and D-dimer are shown in parentheses. Results are presented as mean ± SD*.

* *p-value was calculated using unpaired two-sided t-test*.

§ *p-value was calculated using Fisher exact test*.

*All p-values < 0.05 are significantly different and therefore the values bolded here are significantly different*.

**Figure 2:**
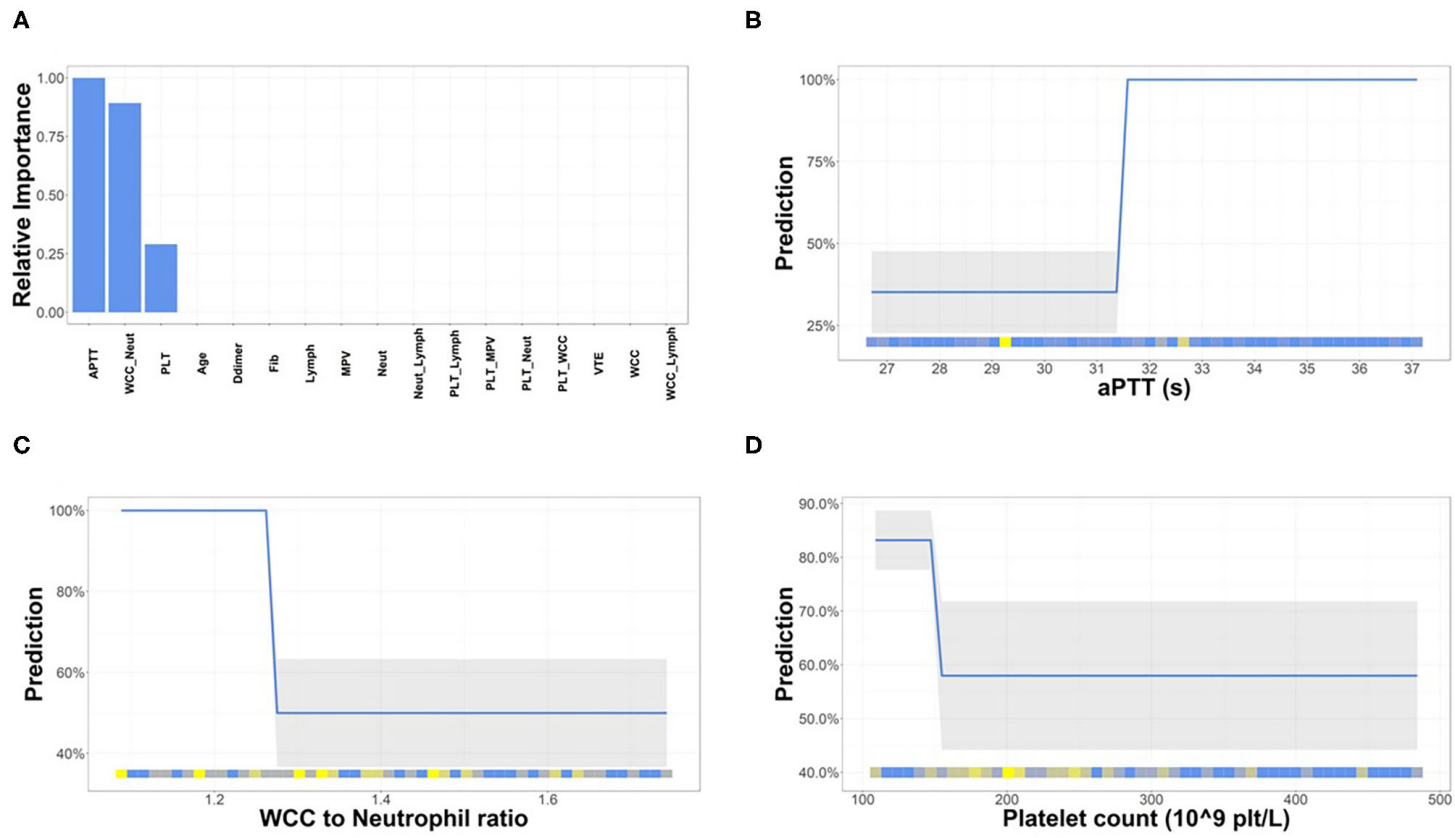
Hematological parameters important for the prediction of severity of COVID-19. **(A)** Of the 20 parameters used for decision modeling, three were deemed the most important for the prediction of severity of COVID-19. The relationship between each parameter [aPTT **(B)**, white cell count (WCC)-to-neutophil ratio **(C)**, and platelet count **(D)**] and predicted target was examined. The partial differentiation (PD) plots show that an aPTT > 31.58 s, WCC-to-neutophil ratio < 1.088, and platelet count < 108.83 × 10^9^ platelets/L are indicative of COVID-19 severity.

To assess the capacity of the clinical hematology workup parameters as predictors of a COVID-19 severity and the need for ICU-level care at the time of a positive COVID-19 test (the day of hospital admission), several models including logistic regression, random forest, and decision tree were considered. A decision tree classifier was chosen as it performs well with missing values and the more complex models such as random forest would lead to overfitting due to our limited sample size. The decision tree model was trained on the patient data from the day of the positive COVID-19 swab (Supplementary Table 1). Parameters with >50% missing data were rejected, and mean imputation was used for other parameters with missing data. The decision model identified aPTT, white cell count-to-neutrophil ratio (WNR), and platelet count as important parameters (Figure 2A). Therefore, only these three (aPTT, WNR, and platelet count) were used in the final decision tree model. The relationships observed between each of the selected parameters and the predicted ICU admission indicate that a person is very likely to require ICU-level care when their aPTT is >31.58 s (normal range: 25.0–36.5 s), WNR is <1.09 and platelet count is <108.83 × 10^9^ platelets/L (normal range: 150–400 × 10^9^ platelets/L) (Figures 2B–D).

**Figure 3:**
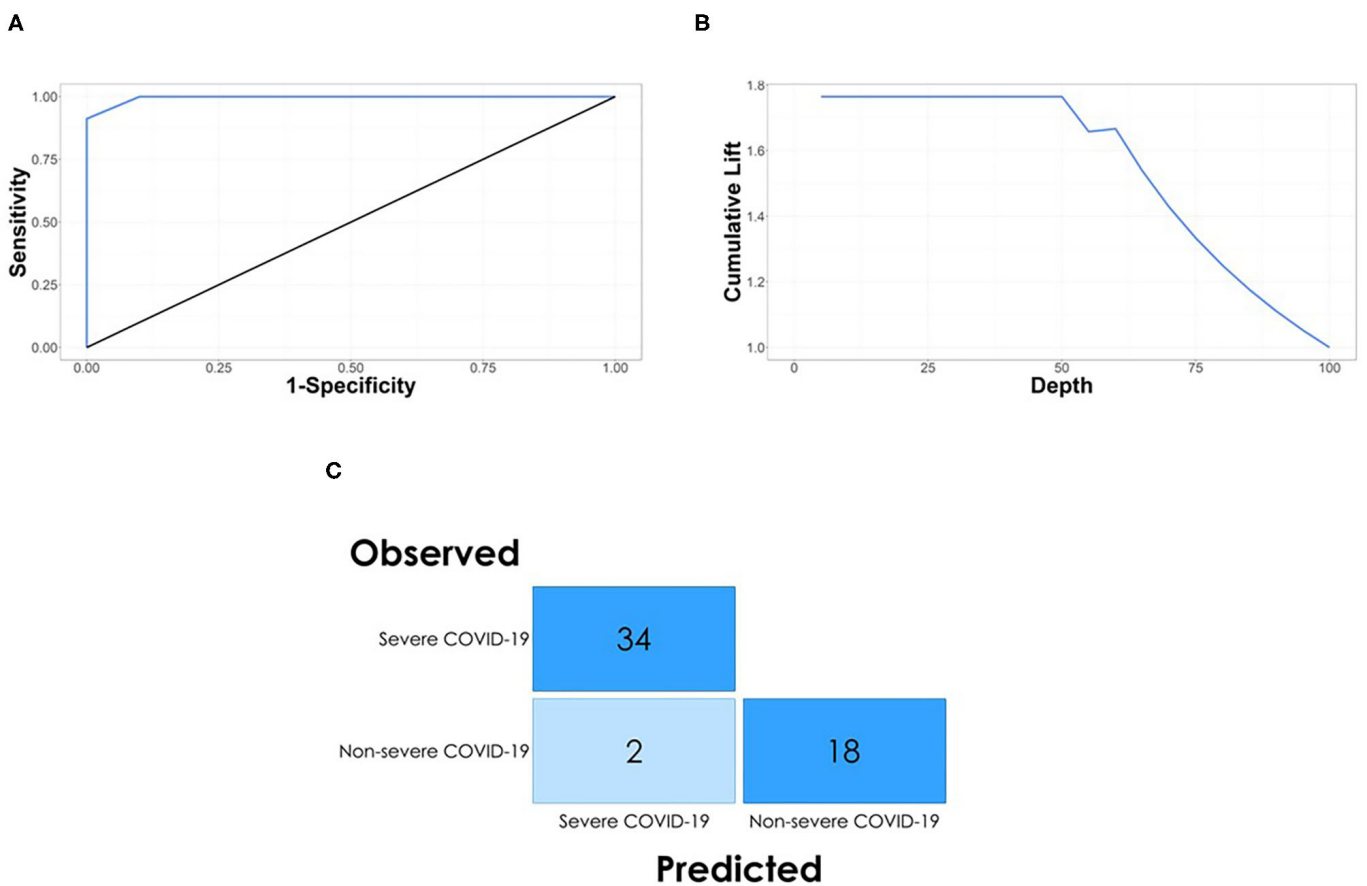
Model performance. **(A)** The ROC curve plot with AUC 0.9956 for the decision tree model indicates that the hematological parameters can be used as predictors for the severity of COVID-19 on the day of positive swab. **(B)** The model achieved a cumulative lift of 1.76 at the 10% quantile, indicating that the model identifies almost two times more severe COVID-19 patients at the day of positive swab than expected with random selection. **(C)** The confusion matrix for the model shows that all of the severe COVID-19 patients and 18 of 20 non-severe COVID-19 patients were correctly classiified by the decision tree model based on three hematological parameters.

The performance of the model was measured using area under ROC (AUROC). Our decision tree model yielded an AUROC of 0.9956 (Figure 3A). The model also achieved a cumulative lift of 1.76 at the 10% quantile, indicating that the model identifies almost two times more severe COVID-19 patients (requiring ICU admission) at the day of positive COVID-19 test (at the time of hospital admission) than expected with random selection (Figure 3B). Finally, the confusion matrix indicates that 96.3% of patients with COVID-19 were correctly allocated into the severe COVID-19 group (requiring ICU admission) and non-severe COVID-19 group (not requiring ICU admission), with only two non-severe COVID-19 patients predicted as severe COVID-19 patients (Figure 3C) at the time of positive COVID swab.

Although our proof-of-concept study involves a small dataset, this result indicates the ability of the model to predict the future need for ICU-level care for COVID-19 patients at the time of positive COVID-19 test (at time of hospital admission). This can be achieved using easily accessible, routinely obtained clinical hematology parameters without requirement for specialized staff training.

## Discussion

In this single-center preliminary study, we demonstrated that simple parameters from a routine hematological workup have the capacity to be useful predictors of ICU admission in COVID-19 patients on the day of positive COVID-19 swab. Our decision tree model demonstrated high sensitivity and specificity with an AUROC of 0.9956 and a misclassification rate of 3.7% at 0.5 cutoff, resulting in an incorrect classification of only 2 out of 20 non-severe COVID-19 patients on the day of positive COVID swab. Recently, several studies describing predictive modeling for COVID-19 have been published, focusing on prediction models for COVID-19 diagnosis (39), COVID-19 severity (40), and patient mortality (41). A common disadvantage of many proposed models is the requirement of detailed patient information including CT scans (27, 28), other imaging data (29, 30), extensive specialist knowledge, that is, APACHE II score (31), additional protein marker tests (32), or extensive patient history and clinical workup (33, 34), and the need for rapid AI-based diagnostic and prognostic system for COVID-19 remains an unmet challenge (18). In contrast to these studies, our proof-of-concept modeling indicated that routinely available hematological parameters such as aPTT, WNR, and platelet count are powerful predictors of severity of COVID-19 in patients at the time of the positive COVID-19 test (admission to the hospital). Therefore, the usage of three standard hematological parameters in a tree-based model would allow for rapid risk stratification of COVID-19 severity in hospitalized patients.

Our findings, indicating that hematological parameters are important for predicting severity of COVID-19, are not unexpected as a substantial body of literature suggests that these are affected by COVID-19. In our model, aPTT at the upper limit of normal or a prolonged aPTT, in addition to thrombocytopenia (decreased platelet count), is predictive of subsequent critical care admission. aPTT is a measurement of coagulation time, specifically the intrinsic activation pathway, and can be used in the diagnosis of coagulopathy such as disseminated intravascular coagulation (DIC) (42). There are several reports documenting small prolongations in COVID-19 patient aPTT (37, 43–46). One study by Bowles et al. noted that aPTT was prolonged in COVID-19 patients despite elevated factor VIII levels, which shortens aPTT (46). These reports notwithstanding, metanalyses are not conclusive on aPTT use as a biomarker (47, 48) and caution is advised on those findings given high levels of heterogeneity among patient cohorts and that aPTT can be affected by other factors such as anticoagulants that are advised in COVID-19 treatment (49, 50).

In addition to aPTT, platelet count was also indicative of severity of COVID-19 in our model, and it has previously been reported to be a marker of COVID-19 severity. In fact, thrombocytopenia has been documented in patients with COVID-19 and was found to be associated with increased risk of in-hospital mortality (44, 51, 52). Interestingly, COVID-19-associated thrombocytopenia is often accompanied by elevated thrombopoietin levels, which we and others have previously reported (53–55). The observation of thrombocytopenia and increased thrombopoietin, while counterintuitive, is reflective of the current literature, and the precise underlying mechanism remains to be elucidated in both COVID-19 and other viral infections. The most likely explanation is that platelet overproduction and consumption may be simultaneously ongoing. Further detailed characterization of this intriguing phenomenon will be valuable.

In contrast to aPTT and platelet count, WNR is a parameter that, following investigations of the literature, has not previously been examined in COVID-19. Several studies have examined neutrophil-to-lymphocyte ratio (NLR) as a biomarker for COVID-19 severity and have proposed a significant prognostic value on NLR for the prediction of disease severity, increased NLR being associated with the severe course of COVID-19 (56–58). The generation of ratios, such as WNR, from routine hematological parameters may provide further routes of investigation and data generation, allowing for more advances in disease prediction modeling.

The small sample size, retrospective data collection, and limited access to clinical baseline characteristics such as pre-existing conditions, medications, and treatments are the main limitations in this study. It is therefore not possible to determine if factors other than disease severity (i.e., capacity within the critical care unit at the time and the “ceiling of care” potentially applied to individual patients based on clinical judgement and the patient's wishes) may have influenced the decision to transfer patients to critical care. It is also possible that some patients with severe COVID-19 may have died prior to transfer to a critical care ward and therefore would not have been included in this analysis. The management of COVID-19 has also evolved in the months since these patients were managed at MMUH and so our data would not take into account the effect of therapies such as corticosteroids in influencing the progression to severe COVID-19. Notwithstanding these limitations, this study presents a potentially valuable prediction model utilizing readily available, routine clinical data.

In summary, severe COVID-19 is characterized by dysregulated inflammation and coagulation activation, and therefore, it is biologically plausible that the hematological parameters included in the prediction model have clinical significance. Our study demonstrates that these routine clinical hematology parameters can identify the patients at risk of developing severe COVID-19 disease. Although not yet in a position to be utilized, the simplicity of the three-branch model based on three hematological parameters is enticing for rapid, wide-scale adoption in clinical practice. It is important to highlight that this is a preliminary study, and the decision tree model presented here must be validated in an extended, prospective cohort of COVID-19 patients. However, once validated, our approach could be easily implemented in clinical setting as it utilizes routine, easily accessible hematological parameters. The advent of effective vaccines and other novel therapeutics will almost certainly lead to reduced COVID-19-related mortality over the coming months. However, until widespread immunity has been achieved on a global scale, it is likely that we will continue to be challenged by the significant burden of disease and healthcare-resource utilization associated with this infection. The availability of prediction models utilizing inexpensive, routine clinical laboratory testing would likely be of significant value to clinicians who continue to be challenged by this disease, particularly in developing countries where healthcare resources are more limited and where access to vaccines may be impeded by the ongoing global demand.

## Data Availability Statement

The original contributions presented in the study are included in the article/Supplementary Material, further inquiries can be directed to the corresponding author/s.

## Ethics Statement

The studies involving human participants were reviewed and approved by Institutional Review Board of the Mater Misericordiae University Hospital (1/378/2077). Written informed consent for participation was not required for this study in accordance with the national legislation and the institutional requirements.

## Author Contributions

PS, HA, SPC, SC, SK, FN, BK, and PM designed the research and wrote the paper. SC, SK, FN, and BK identified and recruited patients to the study. SC, SK, LW, ÁL, and BK compiled the data for all patients recruited to the study. PS, HA, ED, and JC performed data analysis and machine learning. KO'R, AC, BM, SG, NP, and BM contributed to research design and paper writing. All authors contributed to the article and approved the submitted version.

## Conflict of Interest

HA, JC, and ED were employed by company SAS Institute LTD. The remaining authors declare that the research was conducted in the absence of any commercial or financial relationships that could be construed as a potential conflict of interest.

## Supplementary Material

Supplementary Table 1 Hematological parameters of severe and non-severe COVID-19 patients. All hematological parameters (from the time of hospital admission) from patients with severe and non-severe COVID-19 used to build the prediction model.
